# Predictive factors of mortality in damage control surgery for abdominal trauma

**DOI:** 10.1590/0100-6991e-20223390-en

**Published:** 2022-08-25

**Authors:** LUIZA LEONARDI, MARIANA KUMAIRA FONSECA, NEIVA BALDISSERA, CARLOS EDUARDO BASTIAN DA CUNHA, YURI THOMÉ MACHADO PETRILLO, ROBERTA RIGO DALCIN, RICARDO BREIGEIRON

**Affiliations:** 1 - Hospital de Pronto Socorro de Porto Alegre, Residência Médica em Cirurgia Geral e Cirurgia do Trauma - Porto Alegre - RS - Brasil; 2 - Hospital de Pronto Socorro de Porto Alegre, Serviço de Cirurgia Geral e do Trauma - Porto Alegre - RS - Brasil

**Keywords:** Multiple Trauma, Wounds and Injuries, Injury Severity Score, Indicators of Morbidity and Mortality, Risk Factors, Traumatismo Múltiplo, Ferimentos e Lesões, Índices de Gravidade do Trauma, Indicadores de Morbimortalidade, Fatores de Risco

## Abstract

**Introduction:**

damage control surgery (DCS) is well recognized as a surgical strategy for patients sustaining severe abdominal trauma. Literature suggests the indications, operative times, therapeutic procedures, laboratory parameters and intraoperative findings have a direct bearing on the outcomes.

**Objective:**

to analyze the clinical profile of patients undergoing DCS and determine predictors of morbidity and mortality.

**Methods:**

a retrospective cohort study was conducted on all patients undergoing DCS following abdominal trauma from November 2015 and December 2021. Data on subjects’ demographics, baseline presentation, mechanism of injury, associated injuries, injury severity scores, laboratory parameters, operative details, postoperative complications, length of stay and mortality were assessed. A binary logistic regression analysis was performed to determine potential risk factors for mortality.

**Results:**

During the study period, 696 patients underwent trauma laparotomy. Of these, 8.9% (n=62) were DCS, with more than 80% due to penetrating mechanisms. Overall mortality was 59.6%. In the logistic regression stratified by survival, several variables were significantly associated with mortality, including hypotension, and altered mental status at admission, intraoperative cardiorespiratory arrest, need for resuscitative thoracotomy, metabolic acidosis, hyperlactatemia, coagulopathy, fibrinolysis, and severity of the trauma injury scores.

**Conclusion:**

DCS may be appropriate in critically injured patients; however, it remains associated with significant morbidity and high mortality, even at specialized trauma care centers. From pre and postoperative clinical and laboratory parameters, it was possible to predict the risk of death in the studied sample.

## RESUMO

**Introdução:**

a cirurgia de controle de danos (CCD) é estratégia bem definida de manejo cirúrgico para pacientes vítimas de trauma grave. A literatura sugere que as indicações, tempo operatório, medidas terapêuticas adotadas, alterações laboratoriais e achados transoperatórios apresentam impacto direto sobre o desfecho.

**Objetivo:**

analisar o perfil clínico-demográfico dos pacientes submetidos à CCD e identificar fatores preditivos de morbimortalidade na amostra.

**Métodos:**

coorte retrospectiva a partir da análise de prontuários de pacientes submetidos à CCD por trauma abdominal entre novembro de 2015 e dezembro de 2021. As variáveis analisadas incluíram dados demográficos, tempo da admissão, mecanismo do trauma, lesões associadas, escores de trauma, parâmetros laboratoriais, achados cirúrgicos, reposição volêmica e de hemoderivados, complicações pós-operatórias, tempo de internação e mortalidade. Para analisar os fatores de risco para mortalidade, foi utilizada análise de regressão logística binária.

**Resultados:**

no período, foram realizadas 696 laparotomias por trauma abdominal e destas, 8.9% (n=62) foram CCD, sendo mais de 80% por mecanismo penetrante. A mortalidade foi de 59.6%. Na regressão logística estratificada pela sobrevida, diversas variáveis foram associadas à mortalidade com significância estatística, incluindo hipotensão e alteração do estado mental à admissão, parada cardiorrespiratória no transoperatório, necessidade de toracotomia de reanimação, acidose metabólica, hiperlactatemia, coagulopatia, fibrinólise, gravidade dos escores de trauma e necessidade de hemoderivados.

**Conclusão:**

apesar da condução da estratégia de CCD em centro de trauma, a morbimortalidade ainda é elevada. A partir de parâmetros clínicos e laboratoriais pré e pós-operatórios, é possível predizer o risco de evolução para óbito na amostra estudada.

## INTRODUCTION

Damage control surgery (DCS) is based on the premise that patients who have suffered severe abdominal trauma with hemorrhagic shock and physiological compromise are not candidates for laparotomy with definitive treatment of all injuries, as they have little chance of surviving the surgical trauma added to the ongoing physiological derangement. The lethal diamond, composed of hypothermia, metabolic acidosis, coagulopathy, and hypocalcemia is the cascade of events that DCS aims to stop and correct^1^. The strategy is based on the fragmentation of traditional surgery into stages, initially resolving hemorrhage and contamination, and deferring resections and reconstructions for a second intervention, in order to increase the chance of survival^2^ ^-^ ^4^.

The history of this surgical modality arose from the need to control massive hemorrhage in trauma patients, a very frustrating situation for surgeons in the 1980s, when the mortality rate of traditional surgery was up to 90%^5^. The first publication on the topic consists of reports of 31 trauma patients, in which Stone et al.^5^ proposed prioritizing hemorrhagic and contamination control rather than the anatomical repair of all injuries. The authors noted a significant reduction in deaths, even though the postoperative complication rate was 100%. Mortality in the subgroup treated with tamponade was 35%, while in the definitive laparotomy group, this rate reached 93%.

The initial description DCS stages was proposed by Rotondo et al.^6^ as three-phase process. The first consists of controlling bleeding and contamination, with temporary closure of the abdomen. The second, prevention and treatment of hypothermia and correction of coagulopathy and acidosis in the intensive care unit (ICU). The third and last one, indicated after the first 24-72 hours of the first procedure, advocates definitive injury repair, creation of ostomies, definition of the nutritional plan, and closure of the fascia, which may require more than one surgical intervention^3^ ^,^ ^4^ ^,^ ^6^.

Determining factors associated with mortality in patients undergoing DCS is a challenge and several studies have already been published on this topic^7^. However, the literature has not yet established a precise “physiological threshold”, or cutoff points for laboratory values and surgical findings^3^. The aim of this study is to analyze the indications and outcomes of this approach to abdominal trauma in a trauma referral center, as well as to identify potential predictive factors for mortality in the sample.

## METHODS

This is a retrospective cohort study including all patients undergoing exploratory laparotomy for abdominal trauma admitted to the Hospital de Pronto Socorro Municipal de Porto Alegre in a period of six years, between November 2015 and December 2021. After the initial selection, we excluded individuals whose approach was definitive in the first surgery, deaths on the operating table, and cases of DCS for treatment of complications, thus including only patients undergoing DCS at hospital admission.

Variables analyzed included demographics, time from admission to surgery, mechanism of injury, associated injuries, vital signs and Glasgow Coma Scale (GCS) on arrival, trauma scores - Revised Trauma Score (RTS), Injury Severity Score (ISS), Abdominal Trauma Index (ATI), Trauma Injury Severity Score (TRISS) -, laboratory parameters - hemoglobin, arterial blood gas, ionized calcium, prothrombin time (PT), activated partial thromboplastin time (PTT), platelets, fibrinogen, and lactate -, surgical findings, estimated blood loss, volume and blood products transfused, damage control strategy, open abdomen management, postoperative complications, length of stay in the ICU/hospital, and mortality.

All surgeries were performed by surgeons from the General Surgery and Trauma department, with the participation of training residents. The final decision for DCS was at the discretion of the assistant surgeon, due to the absence of a defined institutional protocol.

Statistical analysis was performed using the IBM SPSS^®^ software, version 24.0. The descriptive analysis of was presented in tables of absolute frequency (n) and percentages (%) for categorical variables, and by measures of position and dispersion (mean, median, standard deviation, minimum, and maximum) for continuous ones. The normality of continuous variables was determined by the Kolmorogov-Smirnov test; those with normal distribution were described with measures of mean and standard deviation, and those with asymmetric distribution, with medians and interquartile ranges (IQR; p25 p75). We divided the sample into two groups according to the outcome (survival and death). To compare categorical variables between groups, we used the chi-square or Fisher’s exact tests, as appropriate. Continuous variables with normal distribution were compared using the Student’s t test for independent samples, and those with asymmetric distribution, the Mann Whitney’s U test. The significance level adopted was 5% (p<0.05).

To analyze mortality risk factors, we employed a logistic regression analysis using univariate and multiple models, with input criteria for selecting predictor variables. Those with clinical significance or p<0.10 analyzed separately in the univariate model were included in the multivariate model. The predictor variables were kept in the final model if p<0.05.

The study was carried out based on a secondary database, whose elaboration was approved by the Ethics in Research Committee of the Municipal Health Department of Porto Alegre, under protocol number 3,641,331.

## RESULTS

During the study period, 696 patients underwent exploratory laparotomy for penetrating or blunt abdominal trauma at the Institution, and 11.6% (n=82) required DCS approach at admission. Twenty patients (2.8%) did not survive until the end of the procedure, being recorded as deaths on the operating table and, therefore, excluded from the analysis. Regarding gender distribution, 87% (n=54) of patients were male. The median age was 27 years (5-64, IQR 16). Most were transferred to the hospital by emergency medical services, but 4.8% (n=3) sought the hospital by their own means, while the others were brought by the police.

As for the trauma mechanism, 80.6% (n=50) were penetrating injuries, of which 74.2% (n=46) due to gunshot wounds. Of the 12 (19.4%) victims of blunt trauma, seven (11.3%) were involved in traffic accidents. Associated extra-abdominal injuries were present in 32 (51.6%) patients, most of them in the chest (n=18, 29%) and extremities (n=15, 24.2%). Most patients had tachycardia, hypotension, and normal mental status. The measurement of intra-abdominal pressure and body temperature were not evaluated due to the lack of data in almost all medical records.

The main injuries found intraoperatively were hollow viscera perforations (n=46, 75.4%), solid viscera lacerations (n=33, 53.2%), vascular (n=20, 30.2%), and mesenteric injuries (n=19, 30.6%). Regarding specific organs, the most prevalent were small intestine, in 53.2% (n=33), and colon, in 48.4% (n=30). The major bleeders were solid viscera, mesentery, and large retroperitoneal vessels.

A two-cavity approach was required in 14 patients (22.6%), with laparotomy associated with thoracotomy, extraperitoneal pelvic packing, or both. Intraoperative cardiorespiratory arrest (CRA) occurred in 16 patients (25.8%), and resuscitation thoracotomy was performed in 10 (16%). The estimated time of surgery had a median of 95 minutes (30-300 min, IQR 60). The median time between the initial assessment and arrival at the operating room was 47 minutes (15-600 min, IQR 41).

The median estimated intraoperative bleeding was two liters. The volume replacement approach was uniform, with the administration of tranexamic acid in the first hour in more than 70% of the patients. Crystalloid replacement was greater than two liters in 27.4% (n=23) of cases. Regarding blood products, the median number of transfused bags was four packed red blood cells (PRBC), three units of fresh plasma (FP) intraoperatively, with a FP:PRBC ratio >0.75 in half of cases. However, only 2 patients received fibrinogen and 3 received cryoprecipitate during surgery. Thromboelastography, available at the hospital during a certain period of the study, was performed in 15 (24.2%) patients to guide the correction of coagulopathy.

The median trauma scores reveal the severity profile of the sample, as shown in Table 1. We also observed laboratory alterations such as hyperlactatemia, coagulopathy, anemia, thrombocytopenia, metabolic acidosis, hypocalcemia, and fibrinolysis. As for outcomes, the overall mortality rate was 59.6% (n=37). Of these, 27 patients were classified as early deaths (within 48 hours of surgery). Of all patients, 21 (34%) were discharged and 4 (6.5%) were transferred for further treatment at another institution.

**Table 1:** Clinical-demographic data, initial care, and sample outcomes. Data are reported in n (%) and mean ± standard deviation or median (IQR, p25 p75).

Male	54 (87)
Age	27 (22-38)
Mechanism	
Penetrating	50 (80.6)
Gunshot Wound	46 (74.2)
Stab Wound	4 (6.5)
Blunt	12 (19.4)
Associated injuries	32 (51.6)
Chest	18 (29)
Extremities	15 (24.2)
Face	7 (11.3)
Traumatic brain injury	4 (6.5)
Pelvis	6 (9.6)
Spinal cord	2 (3.2)
Vital signs at admission	
Respiratory rate (irpm)	25 (20-30)
Heart rate (bpm)	113 ± 28
Systolic blood pressure (mmHg)	80 (55-111)
Glasgow Coma Scale (GCS)	14 (12-15)
Trauma indices	
RTS	6.82 (5.35-7.69)
ATI	25 (19-34)
ISS	25 (17-36)
TRISS (%)	92.3 (63.4-97.5)
Shock Index	1.2 (0.9-1.6)
Laboratory	
pH	7.19 (7.08-7.27)
Excess base (mmol/l)	-11 ([-15]-[-5.5])
Bicarbonate (mmol/l)	17 (12.6-19.5)
Hemoglobin (g/dl)	11 (8-12)
Prothrombin time (sec)	53.5 ± 22.8
INR	1.37 (1.15-1.86)
Activated partial thromboplastin time (sec)	35 (29-53.5)
Platelets (x10^3^)	175 (108-223)
Fibrinogen (g/dl)	129 ± 64
Lactate (mg/dl)	5.8 (3.2-11)
Ionized calcium (mmol/l)	1.04 (0.94 - 1.13)
Time until scheduled reintervention (hours)	48 (42 - 72)
Mechanical ventilation time (days)	17 (7 - 27)
Length of ICU stay (days)	54 (87)
Length of stay (days)	23 (10 - 40)
Outcome	29 (17 - 61)
Discharge	
Transfer	21 (34)
Death	4 (6.5)
Early	37 (59.6)
Late	27 (43.5)

INR: International Normalized Ratio; RTS: Revised Trauma Score; ATI: Abdominal Trauma Index; ISS: Injury Severity Score; TRISS: Trauma and Injury Severity Score; ICU: Treatment Unit Intensive.

The median time to scheduled reintervention was 48 hours (20-120 hours, IQR 30). Definitive treatment was possible in the first reintervention in 82.8% (n=29) of the initial survivors, with bowel transit reconstruction prevailing as a second approach, in 62.8% (n=22) of the cases. As for surgical complications, 20 (58.8%) patients required unscheduled intervention, the majority due to gastrointestinal tract fistulas (n=7, 20%) and anastomotic dehiscence (n=10, 28.5%), of whom 10 (28.5%) were treated with an ostomy. Among the initial survivors (>48h after admission), 26 (74.3%) required nutritional support with parenteral nutrition, 13 (37%) progressed to dialysis due to acute renal failure, and 16 (25.8%) underwent tracheostomy.

In the univariate analysis stratified by survival, several variables showed a statistically significant difference between groups. Hypotension, altered GCS, venous vascular injury, need for a two-cavity approach, intraoperative CRA, and trauma scores (except for the ATI and Shock Index), as well as laboratory parameters and the need for PRBC transfusion were more frequent in the death group. Table 2 presents the sample characteristics stratified by outcome.

**Table 2:** Characteristics stratified by survival. Data are reported as n (%), mean ± standard deviation or median (IQR, p25-p75).

	Survival (n=25)	Death (n=37)	p
Age	24 (21-38)	28 (22-38)	ns
Penetrating mechanism	21 (84)	29 (78.4)	ns
Trauma time span	45 (30-70)	50 (30-75)	ns
Heart rate	113 ± 26	114 ± 29	ns
SBP	87 (80-120)	77 (0-103)	.013
GCS	14 (14-15)	14 (8-14)	.024
Associated injuries	10 (40)	22 (59.5)	ns
Venous vascular injury	3 (12)	14 (37.8)	.020
Two-cavity approach	2 (8)	12 (32.4)	.020
Intraoperative CRA	2 (8)	14 (37.8)	.008
Resuscitative thoracotomy	2 (8)	8 (21.6)	ns
RTS	7.11 (6.34-7.84)	5.86 (3.97-7.55)	.015
ATI	24 (16-31)	26 (20-36)	ns
ISS	25 (16-32)	32 (22-41)	.024
TRISS (%)	97.2 (91.5-98.5)	76 (46-95)	<.001
Shock index	1.1 (0.8-1.6)	1.4 (1.0-1.7)	ns
pH	7.24 (7.17-7.3)	7.1 (7.01-7.25)	.008
Base excess	-9.3 (-12.3--6.5)	-13.4 (-19.4--9.9)	.002
Bicarbonate	18.3 (15.5-20)	15.3 (10.3-18)	<.001
Hemoglobin	12 (11-13)	9.5 (7.2-11)	<.001
TP	65 ± 4	44 ± 4	.001
INR	1.19 (1.1-1.48)	1.57 (1.24-1.91)	.001
PTT	32.5 (28.7-36.6)	39.8 (29-102)	.012
Platelets	183 (111-246)	170 (95-210)	.006
	Survival (n=25)	Death (n=37)	p
Fibrinogen	167 ± 11	106 ± 11	<.001
Lactate	3.5 (2.2-6.5)	10.1 (5-15.7)	<.001
Ionized calcium	1.05 (0.99 - 1.14)	0.98 (0.79 - 1.07)	0.032
Crystalloid >2L	5 (22.7)	12 (36.4)	ns
PRBC	3 (2-4)	4 (3-5)	.004
Replacement FP/PRBC <0.75	6 (33.3)	15 (47)	ns
Operative time	90 (60-120)	95 (60-150)	ns
Unscheduled reintervention	7 (28)	7 (18.9)	ns
Reintervenção não programada	7 (28)	7 (18.9)	ns

ns: not statistically significant; CRA: cardiorespiratory arrest; SBP: systolic blood pressure; GCS: Glasgow Coma Scale; PT: prothrombin time; INR: International Normalized Ratio; PTT: activated partial thromboplastin time; FP: fresh plasma; PRBC: packed red blood cells; L: liters.

From the univariate model, the multivariate logistic regression analysis defined as predictive factors of mortality the laboratory alterations in arterial blood gases, blood count, coagulation, and lactate, in addition to the clinical parameters GCS, RTS, ISS, and need for resuscitative thoracotomy (Table 3). The cutoff points above or below which predictive factors were most associated with mortality (50th percentile of the probability curve) were GCS=12, RTS=5.60, ISS=21, BE=-14, HCO3=14.5mmol/l, PT=44.2 seconds, fibrinogen=109.6g/dl, and lactate=6.1mg/dl.

**Table 3:** Binary logistic regression to assess mortality predictors.

	OR (95% CI)	p
GCS	1.278 (1.054 - 1.550)	.013
Systolic blood pressure	1.014 (1.001 - 1.027)	.290
Vascular injury	2.035 (0.605 - 6.799)	.248
Resuscitation thoracotomy	6.947 (1.335 - 36.142)	.021
RTS	1.622 (1.136 - 2.315)	.008
ISS	1.052 (1.004 - 1.101)	.033
TRISS	1.050 (0.998 - 1.005)	.295
pH	4502 (29.760 - 6810)	.001
Base excess	1.286 (1.114 - 1.486)	<.001
Bicarbonate	1.412 (1.165 - 1.711)	<.001
TP	1.066 (1.027 - 1.107)	<.001
Fibrinogen	1.030 (1.014 - 1.046)	<.001
Lactate	1.647 (1.250 - 2.170)	<.001
Ionized calcium	1.011 (0.990 - 1.032)	.319

OR: odds ratio; CI: confidence interval.

There was no statistically significant difference between early (<48h) and late (>48h) deaths regarding trauma mechanism, vital signs at admission, trauma scores, intra-abdominal injuries, and fluid replacement. Operative time was significantly longer among late (150 min; 120-210) compared with early deaths (75 min; 55-120); p=0.005.

## DISCUSSION

The reassessment of DCS indications has been studied over the last few decades. In the review of a North American military trauma center7 published in 2012, some parameters were used to indicate abbreviated surgery, including pH <7.2, laboratory-demonstrated coagulopathy, hollow viscus and vascular injury, hypotension, high Shock Index (>1.2), and need for 4 or more packed red blood cells. Adequate selection for primary definitive surgery in patients with severe physiological impairment will almost inevitably lead to unfavorable outcome or unplanned shortening of the procedure.

In contrast, excessively liberal use (or over-indication) of DCS can prevent the benefits of a single approach among patients with adequate physiological reserve, and expose them to procedures with high potential for morbidity and mortality, such as complications related to the open abdomen and the process of staged surgery itself^3^ ^,^ ^8^. Not all indications for DCS are yet known, however, the patient whose chance of surviving definitive surgery is considerably lower due to the physiological derangement is likely the ideal candidate^3^.

Indications vary from the need of massive transfusion, acidosis, hypothermia, operative time longer than 90 minutes, clinical or laboratory coagulopathy, high lactate, major vascular lesions, and multiple hollow viscera injuries^1^ ^-^ ^3^ ^,^ ^6^ ^-^ ^9^. It is estimated that 10% of patients sustaining severe trauma benefit from this approach, and the sooner the decision is taken, the greater the benefit8. Some authors believe that this decision should be made within the first 15 minutes following hospital admission10. In our series, 8.6% of laparotomies were approached by DCS, a proportion close to that recommended in the literature.

Mortality in DCS is quite variable in the literature. We observed a mortality rate of 59.6%, which is high compared to previously published series. Kapan et al.^10^ reported a rate of 45.8% in a study on risk factors related to DCS with 24 patients. The South African study conducted by Joep et al.^11^, with a sample similar to ours (n=74), observed a surprising mortality rate of 27%. Other international studies report rates ranging from 38.5% to 66%^12^ ^,^ ^13^.

Patients who survive DCS are at increased risk of postoperative complications. Wound infection and anastomotic dehiscence are common due to the high contamination load, and the risk of fistula formation is high in the open abdomen^1^. The overall complication rate found in the present study was 53.2%, very similar to that of Kaplan et al.^10^, of 54.2%. Although DCS reduces mortality compared to definitive surgery in critically ill patients, it still carries high morbidity and mortality, prolonged hospital stays, and significant hospital costs^3^ ^,^ ^4^.

The predictive factors of mortality in the present study were altered mental status, the need for thoracotomy, trauma scores (RTS and ISS), and laboratory abnormalities secondary to metabolic acidosis and coagulopathy. Kaplan et al.^10^ acknowledge pre- and postoperative factors. In the first group, age, base excess, pH, and temperature were statistically significant. In the latter, platelets, INR, packed red blood cell units, Trauma Index, and ISS were also predictive variables. Ordonez et al.^13^ also found a correlation between survival and trauma scores, including ISS, ATI, RTS, and TRISS, body temperature, and laboratory values of pH, fibrinogen, hemoglobin, and PRBC. 

More recently, the literature has highlighted the direct and indirect effects of hypocalcemia on each component of the classic lethal triad, thus proposing low calcium levels as the new arm of the lethal diamond of trauma^1^. Despite the hypocalcemia observed in the death group, ionized calcium values were not statistically significant as risk factors in the multivariate model. As for hypothermia, body temperature below 36ºC for more than four hours is already significant, with reports of 100% mortality if below 32ºC. The lack of temperature data in medical records prevented the analysis of this variable in the present study.

A 2010 Cochrane review article comparing patients with definitive management versus DCS found interesting issues. Of the 1,523 pre-selected articles, no randomized controlled trials were identified. Most publications on the topic come from institutional experiences reported in observational, retrospective, or case studies, probably due to impeding ethical issues^3^ ^,^ ^4^.

The present study has limitations inherent to the retrospective evaluation of medical records, such as inconsistent recording of relevant clinical information. In addition, the assessment of fluid replacement was restricted to the intraoperative period due to the difficulty of accurately measuring it in pre-hospital and ICU settings. The laboratory tests were also not collected at the same time, being the samples collected in some cases at admission, during the operation, or as the first exam in the ICU.

As future perspectives, we suggest the creation of well-defined protocols for the management of these patients, from evaluation in the emergency room to the provision of intensive postoperative care, aiming the standardized care for improved outcomes. The definition of cutoff points for DCS indication should be the subject of prospective studies. Adequate medical record registration by health care teams is required to minimize information loss, reducing registration bias in future studies.

## CONCLUSION

Despite the management at a trauma referral center, the morbidity and mortality of patients undergoing DCS following abdominal trauma is still very high. Based on the present analysis, it is possible to define predictive factors of mortality based on pre- and postoperative clinical and laboratory parameters. The identification and recognition of patients most likely to develop complications or death is essential to better indicate the damage control strategy, as well as to guide the best therapeutic tools during intensive care.
